# Topics of Public Interest

**Published:** 1906-12

**Authors:** 


### TOPICS OF PUBLIC INTEREST.

### A Free University.

AT the forty-fourth annual convocation of the University of the State of New York, held at Albany, October 25-27, 1906, Dr. St. Clair McKelway, of Brooklyn, vice-chancellor of the university, delivered the chancellors' address, the feature of which was a prediction that free university education would ultimately prevail in this state.

### DR. M'KELWAY'S ADDRESS.

MY FRIENDS—The change in our time of meeting will be the first thought in your minds. The Convocation Council made that change thoughtfully. That council comprises representatives of great educational interests in relation with the state. The change of date was reached deliberately. Our midsummer meetings, the memory of many of which is fragrant and inspiring, coincided with other educational occasions which made attendance upon Convocation difficult, and which rendered the presence of not a few eminent educators impossible. Our numbers even at midsummer were rarely small, but the difficulty many had in coming to us was sometimes extreme.

The change of date is, of course, an experiment. The result of this meeting may vindicate the experiment, and we all hope it will. There are no vacations immediately confronting us. Between now and the national holiday soon to occur is enough time for us to deliberate here and depart without a sense of haste to our places of abode. To be sure, an election of significant strenuosity is at hand, but the issues and the candidates to come to judgment at the polls are well known to the people and bear no relation whatever to the work in which we are engaged. Neither the conduct of the State Deparment of Education, nor the record of the Board of Regents, nor the condition of our school system, nor the organization of our state department enters into any platforms or nominations now challenging discussion and nearing decision at the hands of voters. We have no right to apprehend any consequences whatever to our educational system from any result at the polls in this commonwealth in November next, and we certainly have no reason to suppose that any issues or candidates then to be passed on will engage our attention as a convocation during the present meeting.

The reorganisation and the consolidation of our state educational system can now be said to have enough history behind it to justify congratulation and confidence. Where there were two boards there is now but one. Where there were two systems there is now a consolidated system. Where there was conflict there is now concord, and where there was friction or war is now peace.

Advantages can be suggested larger than these. The state has unified its educational system. From the kindergarten all the way through the university the line is straight, plain and strong. The scheme which the parent has in view for the child from when it lisps in numbers to when, again gowned, it marches to graduation, is the scheme which the state has at last enacted. One might think that this simplicity and progressiveness would have been provided at the start. Such, however, was not the case. Education came to the people from the top to the foundation, from the summit to the plain. This was because the original scheme of education contemplated a chosen few rather than the people as a whole. University education is a thousand years older than common school education. But common school education is now the chief solicitude, at least of all enlightened republics, and the provision of it is the bulwark and the glory of the constitutions of all free nations. The first lien on all taxation is held by our common school system. That system is broad enough in many of our states to comprehend the education of the child from the beginning to the end of his scholastic life. I have no objection to the state of New York making its system as broad, as long and as thorough as that. I can conceive of no department of instruction, saving theology, which any new state could not well take in charge, and to which any older state, such as New York, could not safely adjust its educational economy.

A FREE UNIVERSITY EVENTUALLY.

Some of this work of assumption and absorption by our own state must be left to the generations that will succeed our own. We can, however, wisely be more hospitable than hostile to the proposition. We may not expedite it, but we can anticipate it. We would not antagonize private foundations, but we can look forward to the provision of state foundations as broad as those laid in younger commonwealths, and we can confidently expect not a few private foundations of which the conductors are embarrassed and in debt willingly to seek state absorption in the years to come. This would do no violence to specialism or to private initiative. The state is so strong that it can tax all private wealth at will, and so rich that private wealth can provide nothing for its representatives or their children which the state cannot itself provide for the people as a whole. The free school, the free academy, the free college, the latter in parts of our commonwealth, we already have. The free university, with a full complement of professional schools, younger states have and have long had, and this state will eventually have beyond doubt. We may not live to see it, but none of us can live long enough to prevent it, and not a few of us, I hope. will live long enough heartily to welcome it.

The trend of our state toward professional education is a surety and prophecy of this. We allow private institutions to prepare for us law students, but only by state examiners and by state courts can they be admitted to the bar. We allow private institutions to send up to us students in dentistry, in pharmacy, in accountancy, and what not? But only by examinations under state auspices can they be licensed for the practice of their callings. Students of medicine and surgery are similarly educated under private auspices or under chartered institutions allowed by the state, but the medical boards, before which they must finally appear, and by which alone they can be qualified to practise, are provided by state law through our State Board of Regents, as you all well know. All this final state action is a moral justification and a logical prophecy of state initiative in every one of these fields.

When I first became a member of the Board of Regents none of these powers and duties sustained any relation to that board or that board to them. The progressive course which the state pursued brought all these professions under state control in the ultimate, and in that fact is the warrant that the state will provide the power to affect these professions at the initiative. The state will be in no hurry. Time and gravitation will take care of the matter. Both can be trusted to assure their work, and those of us who may indicate or justify our temperament and our conviction, whether by protest or by support, can neither quicken nor stay the forces which in events are as inherent and as indicative as character in the man or in the state.

I think that our present state system of education, which unifies forces, which simplifies mechanism, which harmonizes interests, and which articulates the Educational Department with all the life of all the people. with all the aspirations of all the people, making that department as democratic and as representative as the people are themselves, has been vindicated by the peaceful and progressive results which it has accomplished, or with which it has concurred, and will be so vindicated for as long as our government shall endure among men.

### THE REGENTS ACADEMIC EXAMINATIONS.

At this convocation the State Board of Regents perfected the personnel of the state board of examinations, which is to have charge in the future of the preparation of the question papers for the State Regents' examinations. The appointees are representative of the colleges, the secondary and the elementary schools. They are as follows:

Colleges—Nicholas Murray Butler, president of Columbia University; Rush Rhees, president of the University of Rochester; James R. Day, chancellor of Syracuse University; David W. Hearn, president of the College of St. Francis Xavier, and A. V. V. Raymond, president of Union University, Schenectady.

Secondary Schools—Edward L. Stevens, associate city superintendent, in charge of high schools, New York City; Walter B. Gunnison, principal of Erasmus Hall High School, Brooklyn; Frank Rollins, principal of Stuyvesant High School, Manhattan; Frank D. Boynton, principal of the Ithaca High School, Ithaca, and L. F. Hodge, principal of Franklin Academy, Malone.

Elementary Schools—William Henry Maxwell, superintendent, New York City; Henry P. Emerson, superintendent, Buffalo; A. B. Blodgett, superintendent, Syracuse; Charles E. Gorton, superintendent, Yonkers, and Richard A. Vearing, superintendent, North Tonawanda.

The board consists of twenty members. The Commissioner of Education, the three assistant commissioners and the chief of the examinations division are ex officio members. The appointive members shall serve for four years, but the first appointees for each group shall serve for one, two, three and four years, as designated by the Board of Regents.

The functions of the examinations board shall be to appoint, with the approval of the Commissioner of Education, committees to prepare question papers for state examinations, and to advise with the Commissioner in respect to the form and contents of syllabuses and covering the subjects of study in the elementary and secondary schools.

---

CHOREA CONSIDERED AS CEREBRAL RHEUMATISM. Sir Dyce Duckworth (*British Medical Journal*) after a careful study for a number of years has become convinced that there is more than a commonly accepted intimate association between rheumatism and chorea and that chorea is in fact a cerebral form of rheumatism. His statistics show that there is a family or personal history of rheumatism in 85 per cent. of cases of chorea. He believes that were it possible to obtain accurate histories the per cent. would be larger.—*Wisconsin Medical Recorder.*

